# Nasopharyngeal Carriage and Antimicrobial Susceptibility Profile of *Staphylococcus aureus* among Children under Five Years in Accra

**DOI:** 10.3390/pathogens10020136

**Published:** 2021-01-29

**Authors:** Nicholas T. K. D. Dayie, Mary-Magdalene Osei, Japheth A. Opintan, Patience B. Tetteh-Quarcoo, Fleischer C. N. Kotey, John Ahenkorah, Kevin Kofi Adutwum-Ofosu, Beverly Egyir, Eric S. Donkor

**Affiliations:** 1 Department of Medical Microbiology, University of Ghana Medical School, P.O. Box KB 4236 Accra, Ghana; m4maryosei@yahoo.com (M.-M.O.); japh_opintan@yahoo.com (J.A.O.); pbtetteh-quarcoo@ug.edu.gh (P.B.T.-Q.); fcnkotey@flerholiferesearch.com (F.C.N.K.); ericsdon@hotmail.com (E.S.D.); 2 FleRhoLife Research Consult, P.O. Box TS 853 Accra, Ghana; 3 Department of Anatomy, University of Ghana Medical School, P.O. Box 4236 Accra, Ghana; jahenkorah@ug.edu.gh (J.A.); kadutwum-ofosu@ug.edu.gh (K.K.A.-O.); 4 Department of Bacteriology, Noguchi Memorial Institute for Medical Research, University of Ghana, P.O. Box LG 581 Accra, Ghana; beverly.egyir@gmail.com

**Keywords:** nasopharyngeal carriage, *Staphylococcus aureus*, MRSA, children

## Abstract

This cross-sectional study investigated the *Staphylococcus aureus (S. aureus)* and methicillin-resistant *S. aureus* (MRSA) nasopharyngeal carriage epidemiology in Accra approximately five years post-pneumococcal conjugate vaccines introduction in the country. Archived nasopharyngeal swabs collected from 410 children aged under five years old were bacteriologically cultured. The resultant *S. aureus* isolates were subjected to antimicrobial susceptibility testing and screening for carriage of the *mecA* and *LukF-PV* (*pvl*) genes, following standard procedures. The data obtained were analyzed with Statistical Products and Services Solutions (SPSS) using descriptive statistics and Chi square tests of associations. The isolated bacteria decreased across coagulase-negative Staphylococci (47.3%, *n* = 194), *S. aureus* (23.2%, *n* = 95), Diphtheroids (5.4%, *n* = 22), *Micrococcus* species (3.7%, *n* = 15), *Klebsiella pneumoniae* (3.2%, *n* = 13), *Moraxella* species and *Citrobacter* species (1.5% each, *n* = 6), *Escherichia coli*, *Enterobacter* species, and *Pseudomonas* species (0.9% each, *n* = 2). The MRSA carriage prevalence was 0.49% (*n* = 2). Individuals aged 37–48 months recorded the highest proportion of *S. aureus* carriage (32.6%, 31/95). Resistance of *S. aureus* to the antibiotics tested were penicillin G (97.9%, *n* = 93), amoxiclav (20%, *n* = 19), tetracycline (18.9%, *n* = 18), erythromycin (5.3%, *n* = 5), ciprofloxacin (2.1%, *n* = 2), gentamicin (1.1%, *n* = 1), cotrimoxazole, clindamycin, linezolid, and teicoplanin (0% each). No inducible clindamycin resistance was observed for the erythromycin-resistant isolates. Three (3.2%) of the isolates were multidrug resistant, of which 66.7% (2/3) were MRSA. The *pvl* gene was associated with 59.14% (55/93) of the methicillin-sensitive *S. aureus* (MSSA) isolates, but was not detected among any of the MRSA isolates.

## 1. Introduction

The human nasopharynx and oropharynx are anatomical sites colonized by a wide array of microorganisms—from commensals to potential pathogens—and a higher risk of transmission of these organisms occurs among children [1]. *Staphylococcus aureus*, *Moraxella catarrhalis*, *Haemophilus influenzae*, and *Streptococcus pneumoniae* are examples of these organisms and have been reported to occasionally cause local and disseminated infections—such as pneumonia, bacteraemia, endocarditis, osteomyelitis, meningitis, and skin and soft tissue infections—as a sequel to colonization of these and other anatomical sites [2,3]. Of these commensals, *S. aureus* and *S. pneumoniae* (also called pneumococcus) are the most clinically significant, given their high capacity to cause invasive diseases [4,5,6,7] concurrent with their predisposition to developing multidrug resistance [8,9,10,11,12,13,14]. However, of the two, *S. pneumoniae* is the predominant nasopharyngeal colonizer, and it frequently colonizes children below the age of five; this probably accounts for the high rates of its invasive infections in this population relative to adults [15,16]. As pneumococcal infections are preceded by their carriage, it was deemed necessary to develop and administer vaccines aimed at protecting against pneumococcal carriage [17,18,19]. One of these vaccines, which is currently widely used, is the Pneumococcal Conjugate Vaccine-13 (PCV-13) [17], and its introduction led to a decline in pneumococcal carriage [20].

The introduction of pneumococcal conjugate vaccines, notwithstanding its antecedent positive results recorded in relation to vaccine-captured *S. pneumoniae* serotypes, has been derailed by an upsurge in colonization with non-vaccine *S. pneumoniae* serotypes [21], as well as a perturbation of the nasopharyngeal microbial homeostasis of vaccinated persons and their associates [22,23,24]. A prominently feared perturbation is the insurgence of *S. aureus* in the nasopharynx based on its antagonistic relationship with *S. pneumoniae* [25,26,27,28,29,30], and as a consequence, an escalation of *S. aureus* respiratory tract infections (RTIs) and other diseases [31]. These fears are not unfounded, as *S. aureus*-related acute otitis media has been more frequently reported among PCV-vaccinated individuals [21,25,30]. Moreover, globally, RTIs are recognized key causes of death among adults and children [32]. In addition, *S*. *aureus* is a significant source of RTIs, and its carriage in the nasopharynx is noted as an imperative precursor of its invasive infections [33]. Of principal concern is the substantial proportion of *S. aureus* infections accounted for by the multidrug-resistant methicillin-resistant *S. aureus* (MRSA)—projected at 30.1% of community-acquired and 74.1% of nosocomial infections [34]. Infections with MRSA often occur in tandem with extended hospital stays, increased healthcare costs, and high mortality rates [35,36,37]. One report estimated that MRSA caused 80,000 invasive infections and more than 11,000 deaths in 2011 in the United States of America (USA) [38]. Consequently, PCV-13 introduction could potentially increase the population of reservoirs of *S. aureus*, MRSA, and other multidrug-resistant pathogens, who could serve as a conduit for transmission of these pathogens.

In May 2012, Ghana introduced PCV-13 in the Expanded Immunization Programme as part of efforts to lessen the menace of pneumococcal infections among children aged less than five years [39]; the epidemiology of pneumococcal nasopharyngeal carriage in relation to PCV-13 introduction in Ghana is widely documented [11,15,20,40,41,42]. These reports, however, failed to capture how these PCV-induced dynamics of pneumococcal epidemiology have influenced the nasopharyngeal epidemiology of other colonizers, particularly the *S. pneumoniae*-antagonistic *S. aureus*. It is noteworthy that an in-depth understanding of the PCV-induced evolution of nasopharyngeal *S. aureus* epidemiology is an important step in mounting a robust public health strategy to combat both *S. aureus* and *S. pneumoniae* infections, the brunt of which is borne by children with young immune systems [16,43,44,45]. In Ghana, this is very significant given the several MRSA outbreaks in the country since 2012 [46], the same year of inception of PCV-13 vaccinations in the country. Hence, the aim of this study was to investigate the nasopharyngeal carriage of *S. aureus* and MRSA among children below five years of age in Accra in the conjugate vaccine era, focusing on the prevalence, antibiogram, and Panton-Valentine leukocidin (*pvl*) gene carriage of *S. aureus* and MRSA.

## 2. Methods

### 2.1. Study Site, Design, and Sample Processing

This study was carried out in the Accra metropolis of the Greater Accra Region of Ghana. The metropolis falls within the coastal belt, which has humid and warm climatic conditions. Accra has the highest population density when compared to other districts in the country. According to the 2010 population and housing census, the inhabitants of Accra numbered 1,665,086, representing 42% of the region’s total inhabitants (http://www.statsghana.gov.gh/docfiles). In this study, archived nasopharyngeal swab (NPS) specimens were bacteriologically cultured; these specimens were from a previous cross-sectional survey conducted about five years into the inception of PCV-13 vaccination in the country (from September to December 2016) among 410 children aged under five years [47]. The participants of that study were enrolled from seven randomly selected schools in the metropolis, spanning across the suburbs Kaneshie, Mamprobi, Korle-Gonno, and Palladium (Ashiedu Keteke).

One NPS specimen was collected from each of the study participants by means of FlOQSwabs (Copan Flock Technologies, Italy) swab sticks. Each of these was directly inoculated into its corresponding uniquely labeled vial containing skim milk tryptone glucose glycerol (STGG) transport medium (Oxoid, Basingstoke, UK) and transported to the Department of Medical Microbiology’s research laboratory for storage at −80 °C. The samples were analyzed for the presence of *S. pneumoniae* and archived at −80 °C for use in the current study.

In the current study, which focused on isolating *S. aureus* and bacteria other than *S. pneumoniae*, these archived samples were retrieved from the −80 °C freezer, thawed, brought to room temperature, and vortexed. For each specimen, a loopful was inoculated into sterile tryptic soy broth (Oxoid, Ltd. Basingstoke, UK) and incubated for 48 h at 37 °C. After 48 h of incubation, a loopful of the suspension was plated on sterile blood agar supplemented with 5% sheep blood, MacConkey agar, and mannitol salt agar (Oxoid, Ltd. Basingstoke, UK) and incubated aerobically for 18–24 h.

### 2.2. Isolation and Phenotypic Identification of Bacteria

Identification of the bacteria isolated was done using standard methods, including colony morphology, Gram staining, catalase testing, tube coagulase testing in rabbit-citrate-plasma (Becton and Dickinson ^®^; Heidelberg, Germany), and growth on mannitol salt agar. Gram-positive small to large yellow colonies from mannitol salt agar (Oxoid, Ltd. Basingstoke, UK) were sub-cultured onto blood agar (Oxoid, Hampshire, UK) plates, followed by incubation under aerobic conditions at 37 °C for 18 to 24 h. Catalase testing was performed on large, round, golden-yellow colonies, most of which displayed β-hemolysis on the blood agar plates, to identify staphylococcal isolates. Differentiation of the catalase-positive isolates into *S. aureus* and coagulase-negative Staphylococci was done via coagulase testing. Catalase- and coagulase-positive mannitol-fermenting Gram-positive cocci were identified as *S. aureus* and confirmed as such via *spa* gene screening.

### 2.3. Antimicrobial Susceptibility Testing

Antimicrobial susceptibility tests were performed on the *S. aureus* isolates using Kirby Bauer’s disc diffusion method [28]. A 0.5% McFarland equivalent suspension of each isolate was inoculated on a Muller-Hinton agar (MHA) (Oxoid, Hampshire, UK) plate, followed by incubation at 37 °C for 18–24 h. For each *S. aureus* isolate, within 15 min following the adjustment of the turbidity of the inoculum to 0.5 McFarland suspension using a nephelometer (BD Phoenix Spec ^TM^, Becton, Dickinson and Company, Franklin Lakes, NJ, USA), a sterile cotton swab was dipped into the adjusted suspension and evenly streaked across the entire surface of a sterile and dry Muller-Hinton agar plate with the purpose of obtaining a semi-confluent growth post-incubation.

The antimicrobials used for susceptibility testing included erythromycin (15 μg), clindamycin (2 μg), penicillin G (10 Units), tetracycline (30 μg), cefoxitin (30 μg), cotrimoxazole (1.25/23.75 μg), ciprofloxacin (5 μg), amoxiclav (20/10 μg), teicoplanin (30 μg), linezolid (30 μg), and gentamicin (10 μg), all of BD BBL^TM^ Sensi-Disc Antimicrobial Susceptibility Test Disc. MRSA were screened using cefoxitin (30 μg) discs by the disc diffusion technique [28]. Cefoxitin zones of inhibition greater than or equal to 22 mm and less than 22 mm were considered phenotypically to indicate methicillin-sensitive *S. aureus* (MSSA) and MRSA (confirmation of which was made via screening for *mecA* gene carriage), respectively. The results were interpreted according to the Clinical and Laboratory Standards Institute (CLSI) [28] guidelines. *S. aureus* ATCC 25923 was taken as the positive control strain. Multidrug resistance (MDR) attribute determination was based on the resistance of an organism to three or more different classes of antimicrobials [13]. Isolates that were erythromycin-resistant and clindamycin-sensitive were confirmed for inducible resistance by use of the double disc-zone test (D-zone test). Clindamycin and erythromycin discs were placed at proximities of 15–26 mm from one another on the MHA plates, and these were inspected after 18 h at 37 °C. Flattening of the inhibition zone (D shape) around clindamycin was considered to indicate inducible clindamycin resistance. This test permits the identification of three different phenotypes:

(a). Inducible MLS_B_ (macrolide-lincosamide-streptogramin B) phenotype: iMLS_B_ *S. aureus* isolates show resistance to erythromycin (zone size of ≤13 mm) and sensitivity to clindamycin (zone size of ≥21 mm), giving a D-shaped zone of inhibition around clindamycin with flattening near the erythromycin disc (D test positive).

(b). Constitutive MLS_B_ phenotype: cMLS_B_ *S. aureus* isolates show resistance to both erythromycin (zone size of ≤13 mm) and clindamycin (zone size of ≤14 mm).

(c). Methicillin-sensitive (MS (macrolide–streptogramin)) phenotype: These *S. aureus* isolates show resistance to erythromycin (zone size of ≤13 mm) and sensitivity to clindamycin (zone size of ≥21 mm), but without the D-shaped zone indicative of iMLS_B_ (D test negative).

Additional information on the phenotypic groupings of the D test is presented in Table 1.

### 2.4. Molecular Analysis

Pure *S. aureus* colonies were put in 50 µL of phosphate-buffered saline (PBS) in Eppendorf tubes. The isolates in the Eppendorf tubes were stored at −80 °C until DNA was extracted. DNA was extracted from 95 *S. aureus* isolates using commercial kits from Zymo Research Quick-DNA^TM^ Fungal/Bacterial Mini-prep (Zymo Research Corp., Irvine, CA, USA), following the manufacturer’s instructions.

Multiplex PCR was performed to detect *mecA*, *spa*, and *pvl* genes. This was done following the method described by Larsen et al. [48]. Each PCR reaction contained 10 mM *mecA* primers, 10 mM *spa* primers, and 10 mM *pvl* primers, multiplex PCR Master Mix (New England BioLabs One Tag Quick-Load 2 × Master Mix with Standard Buffer, Beverly, MA, USA), and 1 lL of DNA template preparation. The reaction was performed in an MJ Research PTC-200 Peltier Thermal Cycler. The PCR amplicons were visualized using 2% Agarose, and band size was compared to a 100 bp DNA marker (New England BioLabs, Beverly, MA, USA). The positive controls used were MRSA ATCC 33591, which is positive for *mecA* and *spa* genes, and MSSA ATCC 25923, which is also positive for *spa* and *pvl* genes; nuclease-free water was used as a negative control. Table 2 provides details of the primers used in the multiplex PCR.

### 2.5. Data Analysis

Data were entered into a Microsoft Excel spreadsheet, then imported into Statistical Products and Services Solutions (SPSS) software version 22 and analyzed. Descriptive statistics were used in computing the *S. aureus* carriage prevalence per age group and gender, as well as antibiotic resistance rates. Tests of associations between *S. aureus* carriage and factors such as age and gender were performed using independent-samples Chi-square tests.

### 2.6. Ethical Approval

Ethical approval for this study was obtained from the Ethical and Protocol Review Committee of the College of Health Sciences, University of Ghana (Protocol Identification Number: CHS-Et/M.9-P4.3/2015-2016).

## 3. Results

### 3.1. Demographics of the Study Population 

A total of 410 specimens collected from children under five years of age, who were recruited in a previous study focused on the epidemiology of *S. pneumoniae* nasopharyngeal colonization among the study participants [47], were cultured for the isolation of *S. aureus*. As presented in Table 3, the study participants comprised 51.2% (210/410) males and 48.8% (200/410) females. The mean age of the children sampled was 38.8 months. All 410 participants had been vaccinated with the PCV-13 at ages 6, 10, and 14 weeks from birth, through the Ministry of Health/Ghana Health Service Expanded Programme on Immunization. In the previous study [47], their claims of vaccination were verified by inspection of their vaccination cards. In total, only 61 of the total 410 children were aged ≤2 years.

### 3.2. Nasopharyngeal Staphylococcus aureus Carriage

The *S. aureus* and MRSA nasopharyngeal carriage prevalence rates among the study participants were 23.2% (95/410) and 0.49% (2/410), respectively. Females recorded a higher *S. aureus* carriage prevalence of 24.5% (49/200) than did males, with 21.90% (46/210), but this difference was not statistically significant (χ^2^[1, N = 410] = 0.388, *p* = 0.534). The youngest *S. aureus* carrier was 6 months old, while the oldest was 60 months old. When *S. aureus* carriage was stratified by age group, differences in carriage were statistically significant (χ^2^[4, N = 410] = 15.82, *p* = 0.003), and the age group of 37–48 months (3.1–4.0 years) recorded the highest carriage prevalence of 32.6% (*n* = 31). The two MRSA isolates were found in females aged 36 months (3 years) and 52 months (4 years). The isolation, identification, and characterization of *Streptococcus pneumoniae* present in these samples were reported previously [47]. Table 3 above describes the carriage prevalence and distribution of *S. aureus* per age group and gender, and Table 4 describes the prevalence of pathogens (other than *S. pneumoniae*) isolated from the nasopharyngeal swab samples.

### 3.3. Antimicrobial Susceptibility Profile of the *S. aureus* Isolates

**Figure 1:**
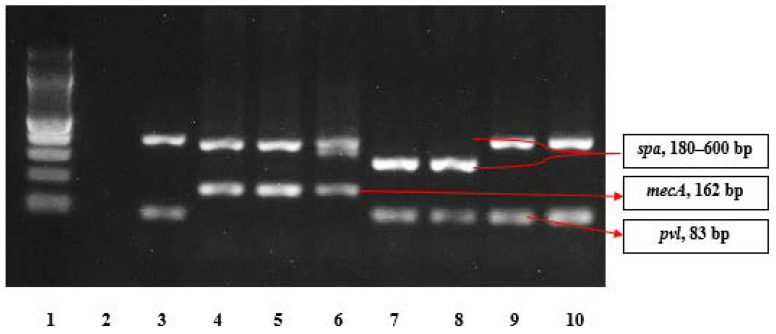
The agarose gel electrophoresis pattern for amplification products of *spa*, *mecA*, and *pvl* genes using multiplex PCR (Lane 1, 100 bp ladder; Lane 2, negative control; Lane 3, positive control for methicillin-sensitive *S. aureus* (MSSA) (ATCC 25923); Lane 4, positive control for methicillin-resistant *S. aureus* (MRSA) (ATCC 33591); Lanes 5–10, *Staphylococcus aureus* isolates).

The resistance of *S. aureus* to the antimicrobials tested decreased across penicillin G (97.9%, *n* = 93), amoxiclav (20%, *n* = 19), tetracycline (18.9%, *n* = 18), erythromycin (5.3%, *n* = 5), cefoxitin and ciprofloxacin (2.1% each, *n* = 2), and gentamycin (1.1%, *n* = 1). All the isolates were susceptible to cotrimoxazole, clindamycin, linezolid, and teicoplanin. The two MRSA isolates were resistant to penicillin G, amoxiclav, tetracycline, and cefoxitin. With the MSSA isolates, resistance was observed against penicillin G (97.8%, *n* = 91), amoxiclav (18.3%, *n* = 17), tetracycline (17.2%, *n* = 16), erythromycin (5.4%, *n* = 5), ciprofloxacin (2.2%, *n* =2), and gentamicin (1.1%, *n* = 1). Multidrug resistance (MDR) was detected in 3.2% (*n* = 3) of the isolates; these exhibited resistance to tetracycline, penicillin, and amoxiclav. Of the three MDR isolates, 66.7% (*n* = 2) were MRSA, and 33.3% (*n* = 1) were MSSA. All the erythromycin-resistant isolates lacked inducible clindamycin resistance and showed the phenotypic D test phenomenon of the macrolide–streptogramin phenotype. The two MRSA isolates were *mec*A gene-positive, but *pvl* gene-negative; 59.14% (*n* = 55) of the MSSA isolates were *pvl* gene-positive; and all 95 *S. aureus* isolates were *spa* gene-positive. The results of the molecular analysis are presented in Figure 1.

## 4. Discussion

The human nasopharynx is colonized by a wide array of microorganisms, including *Staphylococcus aureus*, *Moraxella catarrhalis*, *Haemophilus influenzae*, and *Streptococcus pneumoniae*, of which *S. aureus* and *S. pneumoniae* seem the most clinically significant [2,3,4,5,6,7,12,13,14]. Attempts to control infections of the predominant nasopharyngeal colonizer, *S. pneumoniae*, via vaccination with PCVs have been met with concerns regarding potential disruption of the nasopharyngeal microbial homeostasis to favor *S. aureus* colonization [22,23,24,25,26,27,29,53]. To contribute data to guide deliberations on the matter, our aims were to investigate the nasopharyngeal carriage of *S. aureus* and MRSA (in the context of other nasopharyngeal colonizers) among children below five years of age in Accra approximately five years post-PCV introduction in the country and to report on the prevalence, antibiogram, and *pvl* gene carriage of *S. aureus* and MRSA.

The overall *S. aureus* nasopharyngeal carriage prevalence of 23.2% is comparable to what has been reported previously in Ghana in the nasopharynx of HIV-infected children below five years of age (22.03%) [12] and in the anterior nares of the general population (21%) [54] and children with sickle cell disease (33.3%) [14], but it is heterogeneously lower (44.9%) [13] and higher (8%) [55] than the prevalence among HIV-infected patients post-PCV introduction. It is also comparable to what has been reported for nasopharyngeal carriage in other countries: in The Gambia (20–25.9%) [56,57], Portugal (21.6%) [58], and the Netherlands (23.2%) [25]. However, the post-vaccination carriage prevalence recorded among apparently healthy children in the current study is slightly lower than what was reported in Iran (29.6%) [59], Belgium (34%) [60], Vietnam (30.6%) [61], and The Gambia (30.6%) [62], but higher than that reported in Ethiopia (10.3%) [3]. In the Ethiopian study, Assefa et al. [3] used a relatively smaller sample size of 234, and that might account for the difference in the carriage prevalence reported. As the *S. aureus* nasopharyngeal carriage prevalence recorded in the current study is comparable to the nasal carriage prevalence of the organism recorded in a number of studies conducted in the country post-PCV introduction, it could be discerned that the proportion of *S. aureus* in the nasopharynx seems to be increasing, and this is suggestive that PCV-13 introduction may have played a role, even if infinitesimal. This is because it is the anterior nares that is the ecological niche for *S. aureus*, and not the nasopharynx; hence, the nasopharyngeal carriage prevalence of the organism is expected to be strikingly lower than its carriage in the anterior nares. In fact, PCV-13 introduction seems a reasonable hypothesis to explain the 21% nasopharyngeal carriage prevalence reported in a study by Sampane-Donkor et al. [12] among HIV-infected children post-PCV introduction, despite the fact that HIV infection is a risk factor for *S. aureus* carriage [13]. Even though additional studies would be needed to exhaustively ascertain whether *S. aureus* has become established in the nasopharynx, the extrapolation made is further guided by the report of another study conducted by Adiku et al. [63] prior to PCV-13 introduction in Ghana. The researchers reported a 14.8% nasopharyngeal carriage rate of *S. aureus* among children under five years of age. However, five years after PCV-13 was introduced in Ghana, this study observed a marked increase in *S. aureus* prevalence in the nasopharynx (23.2%). This finding is consistent with what has been reported in areas where PCV-13 has been introduced [29,64]. The phenomenon could be attributable to the removal of the vaccine serotypes of *S. pneumoniae* which regulate the normal flora in the nasopharynx [25,64]. This regulation is carried out through the production of H_2_O_2_, which is bactericidal to *S*. *aureus* [29,65,66]. There are 94 serotypes of *S*. *pneumoniae*, but only 13 serotypes are present in the current vaccine. Past studies have shown that vaccine-type strains are those found to correlate with *S. aureus* carriage, legitimizing the elevated concern that the introduction of the vaccine would indirectly lead to a rise in *S. aureus* carriage and infections [4,5,29,67]. These concerns notwithstanding, as PCV-13 has been instrumental in controlling pneumococcal carriage and fatal infections in this immunologically vulnerable population, it would be premature to call for its withdrawal. Rather, there is a need to brainstorm on how best to fuse its use with curbing *S. aureus* establishment in the nasopharynx.

In this study, high resistance rates to penicillin (98%), amoxiclav (20%), and tetracycline (18.9%) were observed. The high penicillin non-susceptibility observed in this study is consistent with what has been reported previously in Ghana (96–100%) [12,13,14,54,55]. This high prevalence might be due to the fact that HIV-infected individuals and sickle cell disease patients are more exposed to antimicrobials, as well as resistant microbes, owing to their health status [32]. Ampicillin, like its contemporary beta-lactam antibiotic, penicillin, had a high resistance prevalence (100%), which is consistent with what has been reported elsewhere [3]. This observation may be due to the fact that penicillin and ampicillin have been on the market for a long time, are inexpensive, and could easily be obtained over the counter (although being prescription drugs); hence, they may have been misused greatly. In fact, MSSA isolated from urban areas in Africa are known to be highly penicillin-resistant (73.7–100%) [68,69]. The rate of resistance to amoxiclav observed in the current study exceeded what had been reported previously by Risk et al. [70] in The Gambia. The difference could be due to the use of amoxiclav as a first-line presumptively prescribed antibiotic in Ghana [71]. It is noteworthy that there was a marked reduction in tetracycline resistance (18.9%) in this study as compared to a higher resistance rate of 82% reported previously by Donkor et al. [72]. In addition, the 100% susceptibility of *S. aureus* to cotrimoxazole, clindamycin, linezolid, and teicoplanin observed agrees with findings from preceding studies carried out in Ghana [54,73]. This could be due to low or no exposure of the study participants to cotrimoxazole and clindamycin, and also because the drugs linezolid and teicoplanin are not easily accessible and affordable. Sedighi et al. [59] recommended that clindamycin or cotrimoxazole (trimethoprim–sulfamethoxazole) could be used in mild to moderately severe diseases caused by community-acquired MRSA. The double disc diffusion test (D test) was negative for erythromycin-resistant strains in this study, which is in contrast with the report by Sedighi et al. [59]. It is recommended that with erythromycin-resistant strains of *S. aureus*, D testing should always be carried out for the detection of inducible clindamycin resistance.

The 3.2% multidrug resistance (MDR) recorded in this study was below the previously reported 6.0% in Ghana by Egyir et al. [54] and 16.7% by Sampane-Donkor et al. [12] among HIV-infected children (which could be due to frequent exposure to antimicrobial agents as prophylaxes). The low prevalence of MRSA carriage (0.49%) and low proportion of MRSA among *S. aureus* isolates (2.1%, *n* = 2) are consistent with the low carriage rates reported in the country (0–3.4%) [12,13,14,54,55]. This could be ascribed to the low intake of antimicrobial agents like fluoroquinolones (such as levofloxacin or ciprofloxacin, exposure to which enhances MRSA isolation) [26,74] and third-generation cephalosporins (ceftazidime) in the community setting in Ghana, owing to their relatively high costs and characteristic prescription as therapeutic agents for acute infections [54,72]. Cumulative occurrence of MRSA with increasing use of ceftazidime, fluoroquinolones, and co-amoxiclav has also been reported [75].

Sub-Saharan Africa is a known hotspot for *pvl*-carrying MSSA at rates ranging from 17% to 74% [76], thus predisposing populations in these regions to *S. aureus* that could cause polymorphonuclear leucocyte lysis and tissue necrosis. In the present study, 59.14% of the MSSA isolates harbored the *pvl* gene, and this is consistent with the 58% prevalence reported previously in Kumasi, Ghana [73]. Nonetheless, the finding in this study is in sharp contrast to what have been reported in Europe (0.9–1.4%) [60,77]. The two MRSA isolates recovered in this study did not harbor the *pvl* gene, and this mirrors what was reported previously by Egyir et al. [54] in Ghana.

One possible limitation of the current study is that as its parent study primarily focused on *S. pneumoniae* carriage, sampling was done from the nasopharynx, the ecological niche of *S. pneumoniae*, without sampling from the anterior nares (the ecological niche of *S. aureus*), consequently narrowing the robustness with which inferences could be drawn from the data on both nasal and nasopharyngeal *S. aureus* carriage for each study participant. Moreover, a control group was not recruited. However, the extent of these limitations is minimal, as somewhat contemporary data on nasal carriage were available from other studies in the region, which allowed for a measure of extrapolation with the results of the study.

## 5. Conclusions

It is concluded that the nasopharyngeal carriage prevalence of *S. aureus* was high, with a small proportion of these colonizers being MRSA, indicating a possible establishment of *S. aureus* in the nasopharynx of individuals vaccinated with PCV-13. Also, antimicrobial resistance was generally low among the isolates, and inducible clindamycin resistance was absent. Furthermore, a high proportion of the isolates harbored the Panton-Valentine leukocidin gene. The findings of this study highlight the need to study the possible effects of PCV-13 introduction on nasopharyngeal carriage of *S. aureus* among other at-risk populations, such as HIV-infected and sickle cell disease patients.

## Figures and Tables

**Table 1:** Phenotypic groupings and their features in the D test.

Phenotype	Resistance Phenotype	CLI Result	ERY Result	Double Disc Diffusion Test Description
D+	Inducible MLS_B_	S	R	Flattened, D-shaped clear zone around the CLI disc close to resistant ERY disc
D−	MS	S	R	Susceptible zone around the CLI disc and resistant zone around ERY disc
R	Constitutive MLS_B_	R	R	Resistance zones around the CLI and ERY discs
S	No Resistance	S	S	Susceptible clear zones around both discs

S, sensitive; R, resistant; D+, D test positive; D−, D test negative; CLI, clindamycin; ERY, erythromycin; MLS_B_ = macrolide-lincosamide-streptogramin B; MS, macrolide-streptogramin.

**Table 2:** List of primers used for the multiplex PCR detection of *spa*, *pvl*, and *mecA* genes.

Gene	Primer	Sequence (5′–3′)	Size (bp)	Reference
*mecA*	*mecA* P4 *mecA* P7	5′-TCCAGATTACAACTTCACCAGG-3′ 5′-CCACTTCATATCTTGTAACG-3′	162	Oliveira and de Lencastre [49]
*spa* *	*spa* 113F *spa* 1514R	5′-TAAAGACGATCCTTCGGTGAGC-3′ 5′-CAGCAGTAGTGCCGTTTGCTT-3′	180–600	Harmsen et al. [50]
*pvl*	*pvl*F *pvl*R	5′-GCTGGACAAAACTTCTTGGAATAT-3′ 5′-GATAGGACACCAATAAATTCTGGATTG-3′	83	Deurenberg et al. [51]

* Its amplicons are of variable sizes (180–600 bp) [52].

**Table 3:** Carriage prevalence of *Staphylococcus aureus* by age group in children aged ≤5 years attending nursery and kindergarten facilities in Accra.

Age Group (Months)	Age Group (Years)	Number of Children			Carriage Prevalence of *S. aureus*
		Males	Females	Total (%)	
0–12	0–1	2	3	5 (1.2)	3 (3.2%)
13–24	1.1–2	31	25	56 (13.7)	14 (14.7%)
25–36	2.1–3	86	86	172 (42.0)	28 (29.5%)
37–48	3.1–4	70	59	129 (31.5)	31 (32.9%)
49–60	4.1–5	21	27	48 (11.7)	19 (20%)
Total		210	200	410	95

**Table 4:** Bacterial pathogens isolated from the nasopharyngeal swab samples.

Bacterial Pathogen	Number	Prevalence (%)
Coagulase negative Staphylococci	194	47.3
*Staphylococcus aureus*	95	23.2
Diphtheroids	22	5.4
*Micrococcus* species	15	3.7
*Moraxella* species	6	1.5
*Klebsiella pneumoniae*	13	3.2
*Citrobacter* species	6	1.5
*Escherichia coli*	2	0.9
*Enterobacter* species	2	0.9
*Pseudomonas* species	2	0.9

## Data Availability

The data presented in this study are available upon reasonable request from the corresponding author via ntkddayie@ug.edu.gh.
